# Antimutagenic and free radical scavenger effects of leaf extracts from *Accacia salicina*

**DOI:** 10.1186/1476-0711-10-37

**Published:** 2011-12-01

**Authors:** Jihed Boubaker, Hedi Ben Mansour, Kamel Ghedira, Leila Chekir-Ghedira

**Affiliations:** 1 Department of Cellular and Molecular Biology, Faculty of Dental Medicine, Rue Avicenne,Monastir, 5000, Tunisia; 2 Department of Pharmacognosy, Faculty of Pharmacy, Rue Avicenne, Monastir, 5000, Tunisia

## Abstract

**Background:**

Three extracts were prepared from the leaves of *Accacia salicina*; ethyl acetate (EA), chloroform (Chl) and petroleum ether (PE) extracts and was designed to examine antimutagenic, antioxidant potenty and oxidative DNA damage protecting activity.

**Methods:**

Antioxidant activity of *A. salicina *extracts was determined by the ability of each extract to protect against plasmid DNA strand scission induced by hydroxyl radicals. An assay for the ability of these extracts to prevent mutations induced by various oxidants in *Salmonella typhimurium *TA102 and TA 104 strains was conducted. In addition, nonenzymatic methods were employed to evaluate anti-oxidative effects of tested extracts.

**Results:**

These extracts from leaf parts of *A. salicina *showed no mutagenicity either with or without the metabolic enzyme preparation (S9). The highest protections against methylmethanesulfonate induced mutagenicity were observed with all extracts and especially chloroform extract. This extract exhibited the highest inhibitiory level of the Ames response induced by the indirect mutagen 2- aminoanthracene. All extracts exhibited the highest ability to protect plasmid DNA against hydroxyl radicals induced DNA damages. The ethyl acetate (EA) and chloroform (Chl) extracts showed with high TEAC values radical of 0.95 and 0.81 mM respectively, against the ABTS^.+^.

**Conclusion:**

The present study revealed the antimutagenic and antioxidant potenty of plant extract from *Accacia salicina *leaves.

## 1. Background

Exposure to genotoxic chemicals present in food, in the environment, and used in medical treatment can alter the genetic material permanently, and thus may lead cancer [1]. On the other hand, oxidative stress, caused by reactive oxygen species (ROS), is known to cause the oxidation of biomolecules, leading to cellular damage. The tissue injury caused by ROS may include DNA protein and lipids damage [2,3]. Antigenotoxic plant can counter or prevent the adverse effect caused by DNA-damaging chemicals [4]. Drugs obtained from plants have been investigated for the possible presence of mutagenic and/or carcinogenic substances, following the criteria and norms established for synthetic medicines. Fortunately, numerous defense systems protect the cellular macromolecules against oxidation. DNA repair systems take charge of the oxidized bases, the basic site, and the single strand breaks generated by oxidative process. However, cell defences against oxidative stress are also known to decrease through changes in gene expression in response to oxidative stress [5]. The detection and evaluation of the cytotoxic, mutagenic and carcinogenic effects of plant compounds are of fundamental importance in order to reduce the possible risks of these damaging effects. There is an increasing interest in the natural antioxidants contained in the medicinal and dietary plants, which are candidates for the prevention of oxidative damages. Antioxidants from dietary and medicinal plant sources, particularly those containing phenolic compounds, have a significant antioxidant activity [6]. Modern pharmaceutical industries largely take profit of the diversity of secondary metabolites from vegetables for new drug research. This is the case of *Accacia salicina*, The genus *Acacia *is frequently used for the treatment of various illnesses because of their reputed pharmacological effects; published information indicates that *Acacia *has hypoglycemic effects [7], antibacterial, [8] anti-inflammatory activity [9], cestocial [10], spasmogenic and vasoconstrictor activities [11], antihypertensive and antispasmodic activities [12], antiagregation platelet effect [13], as well as an inhibitory effect against hepatitis C virus [14]. The present study was designed to examine antimutagen, antioxidant potenty and oxidative DNA damage protecting activity of plant extract from *Accacia salicina *leaves in relation to their total polyphenol, tannin, sterol and flavonoid content.

## 2. Methods

### 2.1. Chemicals

6-hydroxy-2,5,7,8-tetramethylchroman carboxylic acid (Trolox). Xanthine oxidase (XOD) and 2,2'-Azino-bis-(3-ethylbenzothiazoline-6-sulfonic acid) diammonium salt (ABTS) were obtained from Wako (Osaka, Japan). The mutagen 2- aminoanthracene (2-AA) was purchased from Acros Organics (New Jersey, USA), hydrogen peroxide (H2O2) and Methylmethanesulfonate (MMS) were purchased from Sigma-Aldrich (PO. St Louis, USA). Histidine, biotine and Agar-Agar from Difco (Paris, France). Aroclor 1254 was purchased from Supelco (USA).

### 2.2. Plant materials

*A. salicina *was collected from the Arid Region Institute (IRA) situated in the south east of Tunisia in October, 2003. Botanical identification was carried out by Pr. M. Chaib [15] (Department of Botany, Faculty of Sciences of Sfax). A voucher specimen (AS-10.03) has been kept in the Laboratory of Pharmacognosy, Faculty of Pharmacy of Monastir for future reference. The leaves were shade-dried, powdered, and stored in a tightly closed container.

### 2.3. Extraction procedure and preliminary phytochemical analysis

One hundred twenty grams of powder, from dried leaves, were sequentially extracted in a Soxhlet apparatus (6 h) (AM Glassware, Aberdeen, Scotland, United Kingdom) with petroleum ether, chloroform and ethyl acetate. We obtained the correspondent extracts for each solvant. These types of extracts, with different polarities, were concentrated to dryness and the residues were kept at 4°C. Then, each extract was resuspended in the adequate solvant.

Plant materials were screened for the presence of tannins, flavonoids, coumarins and sterols using the methods previously described by Boubaker *et al. *[16].

The polyphenol content of *A. salicina *leave extracts was quantified by the Folin-Ciocalteau reagent as described by Yuan *et al. *[17]. The Gallic acid (0.2 mg/mL) was used as a standard.

The polyphenol content was expressed according to the following formula:

%Polyphenols=([DOextract×0.2)∕DOGallicacid]∕Extractconcentration×100

However, flavonoid content was determined according to the modified method of Zhishen *et al. *[18]. The Quercetin (0.05 mg/mL) was used as a standard compound. The flavonoïd content was expressed according to the following formula:

%Flavonoids=([DOextract×0.05)∕DOQuercetin]∕Extractconcentration×100

The total sterol content was evaluated as described by Skandrani *et al. *[19]. The sterol content was expressed according to the following formula:

$$\%\text{Sterols}=\left({\text{P}}_{\text{steroids}}∕{\text{P}}_{\text{extract}}\right)\times 100$$

$$\text{Where}\;{\text{P}}_{\text{steroids}}=\left(\text{Mf}-\text{MO}\right)\times 0.25$$

MO: Weight filter (mg), Mf: Weight of filter and precipitate (mg).

The method described by Pearson [20], was used for the determination of tannin content of samples which is evaluated according to the following formula:

%Tannins=(DOextract∕ε×1)∕Extractconcentration×100

where ε; molar extinction coefficient (= l g-1 cm-1) of tannic acid (= 3.27 L g-1 cm-1).

### 2.4. Radical-scavenging activity on ABTS^+•^

An improved ABTS radical cation decolorization assay was used. It involves the direct production of the blue/green ABTS+. chromophore through the reaction between ABTS and potassium persulfate. Addition of antioxidants to the preformed radical cation reduces it to ABTS, to an extent and on a timescale depending on the antioxidant activity, the concentration of the antioxidant and the duration of the reaction. ABTS was dissolved in water to a 7 mM concentration. ABTS^+. ^was produced by reacting ABTS stock solution with 2.45 mM potassium persulfate (final concentration) and allowing the mixture to stand in the dark at room temperature for 12-16 h before use. The ABTS^+. ^solution was diluted with ethanol to an absorbance of 0.7 (± 0.02) at 734 nm. In order to measure the antioxidant activity of extracts, 10 μl of each sample at various concentrations (0.5, 2.5, 4.5, 7.5 and 9.5 mg/ml) was added to 990 μl of diluted ABTS^+• ^and the absorbance was recorded every 1 min. We stop the kinetic reaction after 30 min. Each concentration was analysed in triplicate. The percentage decrease of absorbance at 734 nm was calculated for each point and the antioxidant capacity of the test compounds was expressed as percent inhibition (%). Trolox (6-hydroxy-2,5,7,8-tetramethylchroman- 2-carboxylic acid) is used as a standard in comparison for the determination of the antioxidant activity of a compound. The results are also reported as the Trolox equivalent antioxidant capacity (TEAC), which is the molar concentration of the Trolox giving the same percentage decrease of absorbance of the ABTS as 1 mg/ml of the antioxidant testing extract, at a specific time point [21].

### 2.5. DNA strand scission assay

DNA damage and DNA protecting activity of extracts were detected on pBluescript KS DNA vector. Plasmid DNA was amplified and extracted from *E.coli DH5α *then oxidized with H_2_O_2 _+UV treatment in the presence or absence of the tested extracts and checked on 0.7% agarose in 1X TAE buffer (2 M Tris, 1M Sodium Acetate, 50 mM EDTA, pH = 8) according to the method described by Russo *et al. *[22] with some modifications. In brief, the experiments were performed in a volume of 9 μl in an Eppendorf tube containing 2.34 μg of plasmid DNA, H_2_O_2 _was added to a final concentration of 147 mM with and without 4 μl of extracts at various concentrations.

The reaction was initiated by UV irradiation and continued for 5 min on the surface of UV transilluminator (Bioblock Scientific, TF35 C, France) with intensity of 180W, at 254 nm under room temperature. After irradiation, the mixture was incubated at room temperature during 15 min. Finally, the reaction mixture along with gel loading dye was placed on 0.7% agarose gel for electrophoresis. Untreated pKS DNA was used as a control in each run of gel electrophoresis. Gel was stained with ethidium bromide and photographed with Bio-print (Vilbert lourmat, France).

### 2.6. Bacterial strains

*Salmonella typhimurium *strains TA102 and TA104 which are histidine-requiring mutants, were kindly provided by Pr.I. Felzen, (Universidade do Estado do Rio de Janeiro [UERJ], Rio de Janeiro, Brazil), and maintained as described by Maron and Ames [23]. The genotypes of the test strains were checked routinely for their histidine requirement, deep rough (*rfa*) character, UV sensitivity (*uvrB *mutation) and presence of the R factor. They were stored at -80°C. *S. typhimurium *TA104 and TA102 strains are known to be more responsive to certain mutagens as (2-AA) and (MMS) [23,24].

Strains TA102 and TA104 contain AT base pairs at the *hisG428 *mutant site. The mutation is carried on the multi-copy plasmid pAQ1 in strain TA102 and on the chromosome in strain TA104. The plasmid confers tetracycline resistance, which is a convenient marker to detect the presence of the plasmid. The *hisG428 *mutation is an ochre mutation, TAA, in the *hisG *gene which can be reverted by all six possible base-pair changes; both transitions and transversions. This mutation is also reverted by mutagens that cause oxidative damage [25].

### 2.7. S9 preparation

The S9 microsome fraction is prepared from livers of rats treated with Aroclor 1254 [23]. The components of S9 mix were 8 mM MgCl_2_, 32.5 mM KCl, 5 mM G6P, 4 mM NADP, 0.1 M sodium phosphate buffer (pH = 7.4), and S9 fraction at a concentration of 0.68 mg/ml of mix. The S9 mix was prepared freshly for each assay.

### 2.8. Salmonella-microsome assay

One hundred microliters of an overnight culture of bacteria (cultivated for 16h at 37°C, approximate cell density (2-5) × 10^8 ^cells/ml) and 500 μl of sodium phosphate buffer (0.2 M, pH 7.4 for assay without S9) or 500 μl of S9 mix were added to 2 ml aliquots of top Agar (supplemented with 0.5 mM L-histidine and 0.5 mM D-biotine) containing different concentrations of each extract. The resulting complete mixture was poured on minimal agar plates prepared as described by Maron and Ames [23]. The plates were incubated at 37°C for 48 h and the revertant bacterial colonies of each plate were counted. Negative and positive control cultures gave numbers of revertants per plate that were within the normal limits found in the laboratory. An extract was considered mutagenic if the number of revertants per plate was at least doubled in *S.typhimurium *TA104 and TA 102 strains over the spontaneous revertant frequency [23,26]. Data were collected with a mean ± standard deviation of three plates (n = 3).

### 2.9. Antimutagenicity testing

A modified plate incorporation procedure [27] was employed to determine the effect of all isolates on 2-amino anthracene (2-AA) and Methylmethane sulfonate (MMS) induced mutagenicity. In brief, 0.5 ml of S9 mixture for indirect mutagen (2-AA) and 0.5 ml of phosphate buffer for direct mutagen MMS was distributed in sterilized capped tubes in an ice bath, then 0.1 ml of test compounds and/or 50 μl of mutagen and 100 μl of test compound and 0.1 ml of bacterial culture (prepared as described in mutagenicity test) were added. After vortexing gently and preincubating at 45°C for 30 min, 2 ml of top agar supplemented with 0.05 M L-histidine and D-biotine were added to each tube and vortexed for 3 s. The resulting, entire was overlaid on the minimal agar plate. The plates were incubated at 37°C for 48 h and the revertant bacterial colonies on each plate were counted. The inhibition rate of mutagenicity (%) was calculated relative to those in the control group with the mutagen by the following formula: percent inhibition (%) = [1 - ((number of revertants on test plates - number of spontaneous revertants)/(number of revertants on positive control plates - number of spontaneous revertants))] × 100.

Each dose was tested in triplicate.

### 2.10. Statistical analyses

Data are expressed as mean ± standard deviation from three replicates. The statistical analyses were performed with STATISTICA edition 99 France. Duncan test was used to compare tested compounds vs. positive control. Difference was considered significant when P < 0.05.

## 3. Results

### 3.1. Phytochemical study

The results of our assay on the tested extracts are shown in Table 1. The EA extract showed the presence of significant quantities of tannins, flavonoids and polyphenols. Chl extract showed the presence of coumarins. Whereas, the sterols are detected in a very high quantity in the PE and Chl extracts.

**Table 1:** Phytochemical screening of extracts from Accacia salicina

	PE extract	Chl extract	EA extract
Sterols	++++	++	-
Flavonoids	-	-	+++
Tanins	-	-	+++
Coumarins	-	++	++
polyphenols	-	++	++
Yield (%)	1.90	3.24	2.31

- = not detectable; + = low quantitiy; ++ = average quantitiy +++ = high quantitiy ++++ = very high quantitiy

### 3.2. Determination of Total Polyphenol, Flavonoid, tannins and sterols Contents

The phytochemical study of *A. salicina *extracts showed the presence of various quantities of polyphenols, sterols, tannins and flavonoids (Table 2). The significant content of polyphenols was recorded in EA and Chl extracts. In fact the percentage of total polyphenolic compounds content EA and Chl extracts were 3.31 and 3.62% respectivly. The EP extract showed the presence of an important quantity of sterols 12.5%. The percentage of tannin and flavonoid content in EA extract, were respectively 1.9% and 2.2%.

**Table 2:** Quantitative phytochemical screening of extracts from *Accacia salicina leaves*

Extract content (%)	PE extract	Chl extract	EA extract
Tanins(%)	-	-	1.9 ± 0.01
Flavonoid(%)	-	-	2.2 ± 0.01
Polyphenols (%)	-	3.62 ± 0.008	3. 31 ± 012
sterols(%)	12.5 ± 0.02	5 ± 0.007	2 ± 0.01

(results are represented by the means ± SD of three experiments)

### 3.3. ABTS-scavenging activity

The antioxidant activity of a given compound depends not only on its chemical structure but also on the type of the generated radical it can neutralize. For this reason, we tested the antioxidant potential of the *A. salicina *extracts against more than one radical type. The antioxidant activity measurements of the *A. salicina *extracts, against ABTS^•+^, was expressed as Trolox equivalent antioxidant capacity (TEAC). Since TEAC is a quantification of the effective antioxidant activity of the extract, a higher TEAC would translate a greater antioxidant activity of the tested sample.

The results obtained are summarized in Table 3. EA and Chl extracts exhibited a high antioxidant potential with TEAC values of and 0.81 ± 0.007 0.95 ± 0.004 mM, respectively. EP extract antioxidant capacity were less potent with TEAC values of 0.24 ± 0.008 mM.

**Table 3:** TEAC of ABTS radical formation by *Acaccia salicina *leaf extracts

Extracts ^b^	Concentrations (mg/mL)	Inhibition percentage ^a ^(%)	IC_50_ (mg/mL)	TEAC values mM
	0.05	1.86 ± 1.11		
**PE extract**	0.5	10. 5 ± 2.23		
	2.5	16.7 ± 2.36	-	0.24 ± .008
	4.5	28 ± 1.11		
	7.5	41.5* ± 2.18		

	0.05	2.52 ± 2.03		
**Chl extract**	0.5	19.2 ± 2.53	**1.91 **± 0.08	**0.81 **± 0.007
	2.5	57.8* ± 2.81		
	4.5	72.20* ± 3.82		
	7.5	91* ± 1.65		

	0.05	5.52 ± 2.06		
**EA extract**	0.5	28 ± 1.68		
	2.5	90. 2* ± 2. 12	**1.2 **± 0.04	**0.95 **± 0.004
	4.5	99.28* ± 1.92		
	7.5	100		

TRolox ^c^			**0.26 **± 0.03	**1**
				

^a ^Inhibition of absorbance at 734 nm relative to that of standart ABTS solution

^b ^Values were expressed as means ± standard deviation of three experiments

^c ^positive control

* P < 0.05 compared to negative control without the tested extract by ANOVA followed by student test.

### 3.4. Effect of *Accacia salicina *extracts on pKS plasmid DNA scission induced by hydroxyl radicals

In order to evaluate the ability of the extracts to generate breaks in the phosphodiester bands of DNA, or unlike to protect DNA against the effect of hydroxyl radicals generated by the photolysis of hydrogen peroxide exposed to UV light, plasmid DNA was treated with different concentrations of each extract.

DNA derived from pKS plasmid showed two bands on agarose gel electrophoresis (lane A) the faster moving prominent band which corresponded to the native supercoiled circular DNA and the slower moving band was the open circular form. The UV irradiation of DNA in the presence of H_2_O_2 _resulting the cleavage of native supercoiled circular DNA to give prominent open circular form and a faint linear DNA indicating that OH^. ^generated from UV-photolysis of H_2_O_2 _produced DNA strand scission.

The results showed that the treatment with all extracts doses, did not result in changes in plasmid DNA conformation. These observations suggest that if the extracts cause DNA damage, it is not through direct DNA chain breakage.

**Figure 1:**
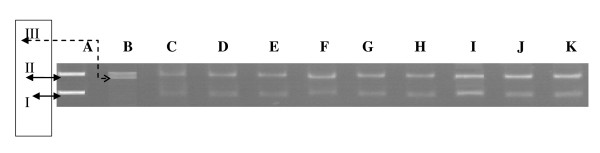
**Electrophoretic pattern of DNA after UV photolysis of H_2_O_2 _in the presence of differentsextracts**. **A **: DNA, **B **: DNA + hydrogen peroxide + UV, **C**: DNA + PE extract (25 μg/assay) + H_2_O_2_, **D **: DNA + PE extract (10 μg/assay) + UV+ H_2_O_2 _, **E **: DNA + PE extract (5 μg/assay) + UV+ H_2_O_2_, **F **: DNA + Chl extract (25 μg/assay) + UV+ H_2_O_2_, **G **: DNA + Chl extract (10 μg/assay) + UV+ H_2_O_2_, **H **: DNA + Chl extract (5 μg/assay) + UV + H_2_O_2_, **I **: DNA + EA extract (25 μg/assay) + UV+ H_2_O_2_, **J **: DNA + EA extract (10 μg/assay) + UV+ H_2_O_2 _, **K **: DNA + EA extract (5 μg/assay) + UV+ H_2_O_2_. **I**: supercoiled form (Sc DNA), **II**: circular-relaxed form (Oc DNA), **III**: linear form (Lin DNA)

In the same way, protective effect of the extracts against OH^. ^induced DNA cleavage, was also studied (Figure 1). All extracts effectively inhibited OH^. ^induced DNA cleavage at the all tested doses.

### 3.5. Mutagenic activity of extracts

No one of the tested extracts induced significant increase of the revertant number of *S. typhimurium *TA102 and *S. typhimurium *TA104 strains, as well with as without metabolic activation (S9) (table 3). It was inferred that neither *A.salicina *extracts nor their metabolits exhibit a mutagenic effect.

### 3.6. Antimutagenicity assay

Doses of 5 and 10 μg/plate of (2-AA), 325 and 130 μg/plate of (MMS) were chosen for the antimutagenicity studies with respectively TA104 and TA102 strains. Since these doses were not toxic and induced 1149 ± 15 (5 μg/plate of 2-AA), and 2144 ± 23 (325 μg/plate of MMS) revertants in *S. typhimurium *TA104. (2-AA) at the concentration of 10 μg/plate induced 652 ± 10 revertants and (MMS) at the concentration of 130 μg/plate induced 1721 ± 24 revertants, in TA102 strain.

Table 4 showed that Chl extract was the most effective in reducing the mutagenicity caused by the direct mutagen MMS, in the TA 104 assay system with respectively inhibition percentages of 64.35% (at a dose of 25 μg/plate) and 55.07% (at a dose of 10 μg/plate). The addition of Petroleum ether extract and Ethyl acetate extract decreased the mutagenicity caused by MMS with respectively 37.66% and 44.54% (at a dose of 25 μg/plate). The inhibition percentage of Petroleum ether extract and Ethyl acetate extract decreased at the different other tested doses.

**Table 4:** Mutagenic effect of different *Accacia salicina *leaf extracts in *S.typhimurium *TA104 and TA102 assay systems in the presence and absence of an exogenous metabolic activation system (S9)

Extracts	Doses (μg/plate)	TA 104		TA 102	
		-S9	+S 9	-S9	+S9
Spontaneous	-	312 ± 8	335 ± 11	244 ± 18	265 ± 25

PC	-	2144 ± 23	1149 ± 15	1721 ± 24	652 ± 10

Petroleum ether extract	25	352 ± 21	375 ± 18	289 ± 11	294 ± 14
	10	348 ± 14	369 ± 17	260 ± 14	284 ± 15

Chloroform extract	5	336 ± 16	361 ± 19	249 ± 13	270 ± 17
	25	336 ± 10	359 ± 21	299 ± 13	304 ± 12
	10	328 ± 11	353 ± 13	249 ± 13	289 ± 15

Ethyl Acetate extract	5	318 ± 10	344 ± 9	242 ± 15	275 ± 19
	25	381 ± 15	363 ± 16	254 ± 22	304 ± 14
	10	333 ± 17	356 ± 20	246 ± 12	294 ± 11
	5	322 ± 25	340 ± 09	242 ± 18	272 ± 19

Positive control (PC): *S. typhimurium *TA104/-S9, MMS (325 μg/plate); *S. typhimurium *TA104/+S9, 2-AA (5 μg/plate); *S. typhimurium *TA102/-S9, MMS (130 μg/plate); *S. typhimurium *TA102/+S9, 2 AA (10 μg/plate).

MMS: Methylmethane sulfonate, 2-AA: 2-amino anthracene

Chl extract showed the most important antimutagenic effect against MMS in the TA102 assay system, in a reverse dose dependent manner (48.75% at a dose of 25 μg/plate) (Table 5). Wheres EA and PE extracts exhibited a maximum inhibition of the (MMS) induced mutagenicity of respectively 41.15% and 32.41% at the tested dose of 25 μg/assay.

**Table 5:** Effect of different extracts from *Accacia salicina *leaves on the mutagenicity induced by MMS in *Salmonella thyphimurium *TA104 and TA102 assay systems without S9

		TA104		TA102	
Extracts	Doses (μg/plate)	Nb revertants	% inhibition of mutagenesis	Nb revertants	% inhibition mutagenesis
Spontaneous		**312 **± 8	-	**234**± 18	-

PC		**2144 **± 23	-	**1721**± 24	-

Petroleum ether extract	**25**	1454 ± 95	**37.66**	1239 ± 73	**32.41**
	**10**	1712 ± 45	**23.58**	1265 ± 42	**30.66**
	**5**	2002 ± 43	7.75	1325 ± 56	2.63

Chloroform extract	**25**	965 ± 99	**64.35**	996 ± 68	**48.75**
	**10**	1135 ± 64	**55.07**	1016 ± 82	**47.41**
	**5**	1266 ± 59	**47.92**	1099 ± 47	**41.82**

Ethyl acetate extract	**25**	1328 ± 58	**44.54**	1109 ± 73	**41.15**
	**10**	1475 ± 98	**36.51**	1195 ± 42	**35.37**
	**5**	1813 ± 54	18.06	1235 ± 56	**32.68**

Positive control (PC): *S. typhimurium *TA104/-S9, MMS (325 μg/plate); *S. typhimurium *TA102/-S9, MMS (130 μg/plate). MMS: Methylmethane sulfonate, 2-AA: 2-amino anthracene.

Chl extracts were highly effective in reducing the mutagenicity caused by the indirect mutagen 2-AA, with 62.77% in the S. typhimurium TA104 assay system and 89% in the S. typhimurium TA102 assay system at a dose of 25 μg/plate (table 6). EA extract was significant effective in reducing the mutagenicity caused by the indirect mutagen 2-AA, with 37% in the S. typhimurium TA104 assay system and 42.2% in the S. typhimurium TA102 assay system at a dose of 25 μg/plate. Whereas no antimutagenic significant effect is detect at the low tested doses in respectivrely the *S. typhimurium *TA102 and *S. typhimurium *TA104 assay systems.

**Table 6:** Effect of different extracts from *Accacia salicina *leaves on the mutagenicity induced by 2-AA in *Salmonella thyphimurium *TA104 and TA102 assay systems in the presence of S9

Extracts		TA104		TA 102	
	Doses (μg/plate)	Nb revertants	% inhibition of mutagenesis	Nb revertants	% inhibition of mutagenesis
Spontaneous		**335 **± 11	-	**235 **± 12	-

PC		**1149 **± 15	-	**652 **± 10	-

Petroleum ether extract	**25**	925 ± 46	**27.51**	520 ± 11	**31.65**
	**10**	934 ± 14	**26.41**	582 ± 8 948 ± 10	16.78
Chloroform extract	**5**	1105 ± 28	5.4		-

	**25**	638 ± 47	**62.77**	281 ± 16	**89**
	**10**	764 ± 38	**47.3**	426 ± 17	**54.2**

Ethyl acetate extract	**5**	881 ± 22	**32.9**	506 ± 7	**35**
	**25**	847 ± 34	**37**	476 ± 5	**42.2**
	**10**	865 ± 52	**34.88**	584 ± 17	16.3
	**5**	1077 ± 19	8.84	652 ± 6	-

PE extract was significant effective in reducing the mutagenicity caused by the indirect mutagen 2-AA, with 27.51% in the S. typhimurium TA104 assay system and 31.65% in the S. typhimurium TA102 assay system at a dose of 25 μg/plate. Whereas no antimutagenic significant effect is detect at the low tested doses in respectivrely the *S. typhimurium *TA102 and *S. typhimurium *TA104 assay systems.

## 4. Discussion

Cellular mechanisms and external factors involved in the production of oxidative stress include the inflammatory response, auto-oxidation of catecholamine, xanthine oxidase activation, pro-oxidants activities of toxins. Scavengers counteract the damaging effects of reactive oxygen species [28]. However, when the balance between these reactive species and antioxidants is altered, a state of oxidative stress results, possibly leading to permanent cellular damage.

Mutations are important early steps in carcinogenesis, therefore, a short term genetic test, such as the *Salmonella*/reversion assay and DNA strand scission assay, have been successfully used for the detection of mutagens/carcinogens, as well as of antimutagens/anticarcinogens [29]. The absence of mutagenicity for PE, Chl and EA extracts of *Accaia salicina *in the two *Salmonella *tested strains TA102 and TA104, with and without (S9) activation system, as well as the absence of phosphodiester band breaks in plasmid DNA at any tested concentration of the different extracts, indicate that DNA does not seem to be revelant target for these extracts [30,31].

In the present experiment we have first investigated the protective role of *A. salicina *extracts against the ABTS^.+^. EA extract revealed a best antiradical activity against ABTS radicals. This should be correlated to their chemical constituents as they are composed by polyphenols and flavonoids in EA extract against ABTS radical. This finding is supported by previous studies reported by Orhan et al. [32] who revealed that the sage polyphenols, including flavone glycosides, were found to display potent antioxidant activities free radicals. In the same context, Hirano et al. [33] and Engelmans et al. [34] demonstrated the flavonoids are able to directly capture the radical species, thus interrupting the radical step of propagation.

This highly activity exhibited by Chl and EA extracts may be correlated to another chemical content. The polyphenolic content appears to function as potent electron and hydrogen atom donors, and therefore should be able to terminate the radical chain reaction by converting the free radicals and the reactive oxygen species to more stable products. Similar observation about the polyphenolic constituents has been reported for several plant extracts such as tea [35,36].

DNA strand scission induced by hydroxyl radicals. Hydroxyl radical is the most reactive radical known in chemistry. It can abstract hydrogen atoms from biological molecules, including thiols, leading to the formation of sulphur radicals capable to combine with oxygen to generate oxysulfur radicals, a number of which damage biological molecules [37]. Althought, both (O2 ^.-^) and H_2_O_2 _are potentially cytotoxic, most of the oxidative damage in biological systems is caused by the OH^.^, which is generated by the reaction between (O2^.-^) and H_2_O_2 _in the presence of metal ions [38]. The UV irradiation of DNA in the presence of H_2_O_2 _resulting the cleavage of Sc DNA to give a prominent Oc DNA and a faint linear DNA indicated that OH^. ^generated from UV photolysis of H_2_O_2 _produced DNA strand scission. The tested extracts showed a significant inhibiting activity against hydroxyl radicals, with the different doses tested. Sterols which are the main constituents of PE and Chl extracts, and which are described as pocessing significant antioxidant activity [39,40] are likely candidates for providing the antigenotoxic effect of these extracts. It is possible that these compounds inhibit the free radicals and ROS produced by oxidation and redox-cycling. We hypothesize that the sterols present in the PE extract, possess different antioxidant properties than those present in the Chl extract, and exhibited a weak scavenging effect than Chl extract against some free radicals. The molecules in the two extracts should have different polarities. These types of compounds; were reported, by many authors, to exhibit an inhibitory effect against some radical systems [41,39,40,42].

In the other hand, the scavenging potential for hydroxyl radicals of Chl extract may be also correlated to its polyphenol content. In fact, polyphenols are an important group of pharmacologically active compounds. They are considered to be the most active antioxidant derivatives in plants [43,44]. However, it has been shown that the phenolic content does not necessary follow the antioxidant activity. Antioxidant activity is generally the result of the combined activity of a wide range of compounds, including phenolics, peptides, organic acids and other components [45].

The chemical components of EA extracts should be better scavenger free radicals. In fact both of them contain flavonoids which are described by Rice-Evans [46] and Kumar and Chattopadhyay [47], as effective hydrogen donors, making extracts potent antioxidants. These compounds should also act through a variety of mechanisms including scavenging of ROS [48]. We believe that the presence of such chemicals in the EA extract explain the important O2^.- ^scavenging effect of both extracts. In a study employing a non-enzymatic system to generate superoxide radicals [49], it was shown that flavonoids are able to scavenge O2^.- ^[50].

As far as antioxidants has attracted much interest with respect to their protective effect against free radical damage that may be the cause of many diseases including cancer, antimutagenic activity of *A. salicina *extracts was investigated in the present study.

In the present experimental conditions Chl extract was an effective antimutagen against two different types of genotoxic compounds direct and indirect acting mutagenes suggesting that the extracts can act through various mechanisms. They reduced frameshift mutagenicity induced by (2-AA) and (MMS), an direct-indirect acting agent, suggesting that they could interfere with the metabolic activation of promutagens, by functioning as blocking agents [51]. The *P*-450 enzyme system catalyzes the formation of *N*-hydroxy derivatives, such as *N*- ydroxy-2- aminoanthracene (a metabolite that interacts with DNA). Thus, an alteration in the function of the enzyme may result in altered reaction rates and differential pathways of the metabolism of mutagens and carcinogens. In some cases, this modification provides protection against chemically induced mutagenesis. In fact, this effect is known to play a role in the antimutagenicity of some plant extracts [51,52]. These data agree with the knowledge that anticarcinogenicity of polyphenols contributes to block the formation of carcinogen [53]. However, the Chl extract may also directly protect DNA from the electrophilic metabolites of the mutagen given that favonoids provide strong nucleophilic centers, which enables them to react with electrophilic mutagens and form adducts that may result in the prevention of genotoxic damage [54]. The observed antimutagenicity of the Chl extract in the TA102 strain (sensitive to oxidative damage) and TA104 strain is congruent with its strong antioxidant capacity. This result suggests that consumption of the studied plants could be an alternative for reducing genotoxic damage induced by free radicals. The observed antioxidant potential could be related to the presence of polyphenolic compounds [55-57]. Polyphenols, which are widely distributed in the plant kingdom and are present in considerable amounts in fruits, vegetables, spices, medicinal herbs, and beverages, have been used to prevent many human diseases, such as diabetes, cancers, and coronary heart diseases [58]. The biological activities of polyphenols in different systems are believed to be due to their redox properties, which can play an important role in absorbing and neutralizing free radicals, quenching singlet and triplet oxygens, or decomposing peroxides [59].

Sterols, wich are the main constituents of PE extract, seem to be most likely candidates for providing the observed antimutagenic activity of this extract [41].

Protective effect of PE, Chl and EA extracts against the tested mutagens may probably adsorb the mutagen in a way similar to the carcinogen adsorption which has been associated with pyrrole pigments, such as hemin and chlorophyllin [60,61].

The differents antimutagenic activity of Chl than EA and PE extracts could be explained by the antioxidant activity is often the result of the combined activity of a wide range of compounds, including phenolics, peptides, organic acids and other components [62] and to the different sensibilities of the two strains towards a given compound or complex [63].

## 5. Conclusion

In conclusion, the present study demonstrates that extracts of *A. salicina *possesses potent antioxidant and antimutagenic activities. These extract is capable of protecting against oxidative DNA damage. Further investigations on testing their *in vivo *activities and on isolation and characterization of the active compounds responsible for the antioxidant capacity of *A. salicina *leaf extracts are under way in our laboratory.

## Conflict of interests statement

The authors declare that they have no competing interests.

## Authors' contributions

JB: Was responsible for the conception and design, testing and data acquisition, analysis and data interpretation and drafted the manuscript. HBM: Was responsible for the conception and design, testing and data acquisition, analysis and data interpretation and drafted the manuscript. The two first authors are contributed equally in this work

KG: made substantial contribution to conception and revised it critically for important intellectual content. LCG: made substantial contribution to conception and revised it critically for important intellectual content. All authors read and approved the final manuscript.

## References

[B1] Bartolome A Mandap K David KJ Sevilla F III Villanueva J SOS-red fluorescent protein (RFP) bioassay system for monitoring of antigenotoxic activity in plant extract Biosens Bioelectron 2006 21 2114 2120 10.1016/j.bios.2005.10.009 16321516

[B2] Fraga CG Arias RF Llessuy SF Koch OR Boveris A Effect of vitamin E and seleniumdeficiency on rat liver chemiluminescence Biochem J 1987 2 383 386 10.1042/bj2420383 PMC1147716 3593258

[B3] ramarathan N OSAWA NT Namiki M Tashiro T J SCI fOOD Agri 1986 37 719 10.1002/jsfa.2740370803

[B4] Ben Sghair M Boubaker J Naffati Aicha Limem I Skandrani I Bhouri W Bouhlel I Kilani S Chekir Ghedira L Ghedira K Antimutagenic and Antioxidant Potentials of Teucrium Ramosissimum Essential Oil Chem Biodiverity 2010 7 1 10 10.1002/cbdv.200900185 20658663

[B5] Johnson FB Sinclair DA Guarente L Molecular biology of aging Cell 1999 2 291 302 10.1016/s0092-8674(00)80567-x 9988222

[B6] Gulcin I Bursal E Sehitoglu MH Bilsel M Goren AC Polyphenol contents and antioxidant activity of lyophilized aqueous extract of propolis from Erzurum, Turkey Food and Chem Toxicol 2010 48 2227 2238 10.1016/j.fct.2010.05.053 20685228

[B7] Wadood A Wadood N Wahid Shah SA Effects of *Acacia arabica *and *Caralluma edulis *on blood glucose levels of normal and alloxan diabetic rabbits J Pak Med Assoc 1989 39 208 212 2509753

[B8] Sotohy SA Sayed AN Ahmed MM Effect of tannin-rich plant (*Accacia nilotica*) on some nutritional and bacteriological parameters in goats Deutsche Tierarztliche Wochenschrift 1997 104 432 435 9394540

[B9] Dafallah AA Al-Mustapha Z Investigation of the anti-inflammatory activity of *Acacia nilotica *and *Hibiscus sabdariffa* Am J Chinese Med 1996 24 263 269 10.1142/S0192415X96000323 8982438

[B10] Ghosh NK Babu SP Sukul NC Ito A Cestocidal activity of *Acacia auriculiformis* J of Helmintol 1996 70 171 172 10.1017/S0022149X00015340 8960214

[B11] Amos S Akah PA Odukwe CJ Gamaniel KS Wambede C The pharmacological effects of an aqueous extract from *Acacia nilotica *seeds Phytother Res 1999 13 683 685 10.1002/(SICI)1099-1573(199912)13:8<683::AID-PTR534>3.0.CO;2-X 10594939

[B12] Gilani AH Shaheen F Zaman M Janbaz KH Shah BH Akhtar MS Studies on hypertensive and antispasmodic activities of methanol extract of *Acacia nilotica *pods Phytother Res 1999 14 510 516 10.1002/(sici)1099-1573(199912)13:8<665::aid-ptr563>3.0.co;2-t 10594935

[B13] Shah BH Safdar B Virani SS Nawaz Z Saeed SA Gilani AH The antiplatelet aggregatory activityof *Accacia nilotica *is due to blockage of calcium influx through membrane calcium channels General Pharmacol 1997 29 251 255 10.1016/s0306-3623(96)00413-2 9251908

[B14] Hussein G Miyashiro H Nakamura N Hattori M Kakiuchi N Shimotohno K Inhibitory effects of Sudanese medicinale plant eextracts on hepatitis C virus (HCV) Phytother Res 2000 14 510 516 10.1002/1099-1573(200011)14:7<510::AID-PTR646>3.0.CO;2-B 11054840

[B15] Cheib M Boukhris M Flore succinct et illustré des zones arides et sahariennes de Tunisie 1998 49 43 44

[B16] Boubaker J Skandrani I Bouhlel I Ben Sghaier M Neffati A Ghedira K Chekir-Ghedira L Mutagenic, antimutagenic and antioxidant potency of leaf extracts from *Nitraria retusa* Food and Chem Toxicol 2010 48 2283 2290 10.1016/j.fct.2010.05.061 20510330

[B17] Yuan VY Bone DE Carrington F Antioxidant activity of dulse (*Palmaria palmata*) extract evaluated in vitro Food Chem 2005 91 485 494 10.1016/j.foodchem.2004.04.039

[B18] Zhishen J Mengcheng T Jianming W The determination of flavonoid contents in mulberry and their scavenging effects on superoxide radicals Food Chem 1999 64 555 559 10.1016/S0308-8146(98)00102-2

[B19] Skandrani I Ben Sghaier M Neffati A Boubaker J Bouhlel I Kilani S Mahmoud A Ghedira K Chekir-Ghedira L Antigenotoxic and free radical scavenging activities of extracts from *Moricandia arvensis* Drug Chem Toxicol 2007 30 361 382 10.1080/01480540701522494 17934925

[B20] Pearson D The Chemical Analysis of Foods 1976 7 London: Churchill Livingstone 572

[B21] Gulcin I Antioxidant properties of resveratrol: A structure-activity insight Innovative Food Science and Emerging Technologies 2010 11 210 218 10.1016/j.ifset.2009.07.002

[B22] Russo A Acquaviva R Campisi A Srrenti V Di Giacomo C Virgata G Bioflavonoids as antiradicals, antioxidants and DNA cleavage protectors Cell Biol Toxicol 2000 16 91 98 10.1023/A:1007685909018 10917564

[B23] Maron DM Ames BN Revised methods for the *Salmonella *mutagenicity test Mutat Res 1983 113 173 215 6341825 10.1016/0165-1161(83)90010-9

[B24] Nelson GM Swank AE Brooks LR Bailey KC George SE Metabolism, microflora effects, and genotoxicity in haloacetic acid-treated cultures of rat cecal microbiota Toxicology Science 2001 60 232 241 10.1093/toxsci/60.2.232 11248134

[B25] Mortelmans K Zeiger E The Ames *Salmonella*/microsome mutagenicity assay Mutat Resear 2000 455 29 60 10.1016/S0027-5107(00)00064-6 11113466

[B26] Marques RCP De Medeiros SRB Da Silva Dias C Barbosa-Filho JM Agnez-Lima LF Evaluation of the mutagenic potential of yangambin and of the hydroalcoholic extract of *Ocotea duckei *by the Ames test Mutation Research 2003 536 117 120 12694751 10.1016/s1383-5718(03)00040-8

[B27] Lee KT Sohn IC Park HJ Kim DW Jung GO Park KY Essential moiety of antimutagenic and cytotoxic activity of *hederagenin monodesmosides *and *bidesmosides *isolated from the stem bark of *Kalapanox pictus* Planta Medica 2000 66 329 332 10.1055/s-2000-8539 10865448

[B28] Uliasz TF Hewett SJ A microtiter trypan blue absorbance assay for the quantitative determination of excytotoxic neuronal injury in cell culture Journal of Neuroscience Methods 2000 100 157 163 10.1016/S0165-0270(00)00248-X 11040379

[B29] Rausher R Edenharder R Platt KL *In vitro *antimutagenic and *in vivo *anticlastogenic effects of carotenoids and solvent extracts from fruits and vegetables rich in carotenoids Mutation Research 1998 413 129 142 9639691 10.1016/s1383-5718(98)00017-5

[B30] Kilani S Ben Ammar R Bouhlel I Abdelwahed A Hayder N Mahmoud A Ghedira K Chekir-Ghedira L Investigation of extracts from (Tunisian) *Cyperus rotundus *as antimutagens and radical scavengers Environmental Toxicology and Pharmacology 2005 3 478 484 10.1016/j.etap.2005.05.012 21783629

[B31] Ben Ammar R Bouhlel I Valenti K Ben Sghaier M Kilani S Mariotte AM Dijoux-Franca MG Laporte F Ghedira K Chekir-Ghedira L Transcriptional response of genes involved in cell defense system in human cells stressed by H2O2 and pre-treated with (Tunisian) *Rhamnus alaternus *extracts: Combination with polyphenolic compounds and classic *in vitro *assays Chemico-Biological Interactions 2007 168 171 183 10.1016/j.cbi.2007.04.002 17512922

[B32] Orhan I Kartal M Naz Q Ejaz A Yilmaz G Kan Y Konuklugil B Sener B Choudhary MI Antioxidant and anticholinesterase evaluation of selected Turkish Salvia species Food Chem 2007 103 1247 1254 10.1016/j.foodchem.2006.10.030

[B33] Hirano R Sasamoto W Matsumoto A Itakura H Igarashi O Kondo K Antioxidant ability of various flavonoids against DPPH radicals and LDL oxidation J Nutr Sci Vitaminol 2001 47 357 362 10.3177/jnsv.47.357 11814152

[B34] Engelmann MD Hutcheson R Cheng IF Stability of Ferric Complexes with 3-Hydroxyflavone(Flavonol), 5,7-Dihydroxyflavone (Chrysin), and 3', 4'- Dihydroxyflavone J Agric Food Chem 2005 53 2953 2960 10.1021/jf048298q 15826045

[B35] Yen GC Chen HY Antioxidant activity of various tea extracts in relation to their antimutagenicity J Agric Food Chem 1995 43 27 32 10.1021/jf00049a007

[B36] Amorowicz R Pegg RB Rahimi-Moghaddam P Barl B Weil JA Free radical scavenging capacity of selected plant species from the Canadian prairies Food Chem 2004 84 551 562 10.1016/S0308-8146(03)00278-4

[B37] Dahl MK Richardson T Photogeneration of superoxide anion in serum of bovine milk and in model systems containing riboflavin and amino acid Journal of Dairy Science 1978 61 400 407 10.3168/jds.S0022-0302(78)83613-3

[B38] Halliwell B Reactive oxygen species in living systems: source, biochemistry, and role in human disease Annal Journal Medical 1991 91 14 25 10.1016/0002-9343(91)90279-7 1928205

[B39] Argolo ACC Sant'Ana AEF Pletsch M Coelho LCBB Antioxidant activity of leaf extracts from *Bauhinia monandra* Bioresource Technology 2004 95 229 233 10.1016/j.biortech.2003.12.014 15246449

[B40] Ben Mansour H Boubaker J Bouhlel I Mahmoud A Bernillon S Ben Chibani J Ghedira K Chekir-Ghedira L Antigenotoxic activities of crude extracts from *Acacia salicina *leaves Environmental and Molecular Mutagenesis 2007 48 58 66 10.1002/em.20265 17177209

[B41] Bouhlel I Valenti K Kilani S Skandrani I Ben Sghaier M Mariotte AM Dijoux-Franca MG Ghedira K Hininger-Favier I Laporte F Chekir-Ghedira L Antimutagenic, antigenotoxic and antioxidant activities of *Acacia salicina *extracts (ASE) and odulation of cell gene expression by H2O2 and ASE treatment TIV 2008 22 1264 1272 10.1016/j.tiv.2008.04.008 18515041

[B42] Shon MY Choi SD Kahng GG SH N Sung NJ Antimutagenic, antioxidant and free radical scavenging activity of ethyl acetate extracts from wite, yellow and red onions Food Chemi Toxicol 2004 42 659 666 10.1016/j.fct.2003.12.002 15019191

[B43] Edenharder R Grunhage D Free radical scavenging abilities of flavonoids as mechanism of protection against mutagenicity induced by tertbutyl hydroperoxide or cumene hydroperoxide in *Salmonella typhimurium *TA102 Mutation Research 2003 540 1 18 12972054 10.1016/s1383-5718(03)00114-1

[B44] Park KY Jung GO Lee KT Choi J Choi MY Kim GT Jung HJ Park HJ Antimutagenic activity of flavonoids from the heartwood of *Rhus verniciflua* J Ethnopharmacol 2004 90 73 79 10.1016/j.jep.2003.09.043 14698512

[B45] Gallardo C Jimenez L Garcia-Conesa MT Hydroxycinnamic acid composition and *in vitro *antioxidant activity of selected grain fractions Food Chemi 2006 99 455 463 10.1016/j.foodchem.2005.07.053

[B46] Rice-Evans CA Miller NJ Bramley PM Pridham JB The relative antioxidant activity of plant derived polyphenolic flavonoids Free Rad Res 1995 22 375 383 10.3109/10715769509145649 7633567

[B47] Kumar A Chattopadhyay S DNA damage protecting activity and antioxidant potential of pudina extract Food Chem 2006 100 1377 1384

[B48] Vundac VB Brantner AH Plazibat M Content of polyphenolic constituents and antioxidant activity of some Stachys taxa Food Chem 2007 104 1277 1281 10.1016/j.foodchem.2007.01.036

[B49] Schuldta EZ Farias MR Ribeiro-do-Vallea RM Ckless K Comparative study of radical scavenger activities of crude extract and fractions from *Cuphea carthagenensis *leaves Phytomed 2004 11 523 529 10.1016/j.phymed.2003.05.005 15500264

[B50] Shu-Jing Wu Lean-Teik Ng Antioxidant and free radical scavenging activities of wild bitter melon (*Momordica charantia *Linn. var. abbreviata Ser.) in Taiwan LWT Food Sci Technol 2008 41 323 330 10.1016/j.lwt.2007.03.003

[B51] Horn RC Ferrao VVM Antimutagenic activity of extracts of natural substances in the *Salmonella*/microsome assay Mutagenesis 2003 18 113 118 10.1093/mutage/18.2.113 12621065

[B52] Zani F Cuzzoni MT Daglia M Benvenuto S Vampa G Mazza P Inhibition of mutagenicity in *Salmonella typhimurium *by *Glycyrrhiza glabra *extract, glycyrrhizinic acid,18 alpha-, and 18 beta-glycyrrhetinic acids Planta Medica 1993 59 502 507 10.1055/s-2006-959748 8302947

[B53] Lin JK Liang YC Cancer Chemoprevention by Tea Polyphenols Proceedings of the National Science Council. ROC(B) 24, No. 1 2000 1 13 10786933

[B54] Marnewick LJ Gelderblom CAW Joubert E An investigation on the antimutagenic properties of South African teas Mutation Research 2000 471 157 166 11080671 10.1016/s1383-5718(00)00128-5

[B55] Galati G O'Brien JP Potential toxicity of flavonoids and others dietary phenolics: significance for their chemopreventive and anticancer properties Free Radical Biology and Medicine 2004 37 287 283 10.1016/j.freeradbiomed.2004.04.034 15223063

[B56] Okawa M Kinjo J Nohara T Masateru O DPPH (1,1-diphenyl-2-picrylhydrazyl) radical scavenging activity of flavonoids obtained from some medicinal plants Biological Pharmaceutical Bullettin 2001 24 1202 1205 10.1248/bpb.24.1202 11642334

[B57] Payet B Sing CS Smadia J Assessment of antioxidant activity of cane sugars by ABTS and DPPH radical scavenging assays: determination of their polyphenolic and volatile constituents Journal of Agricultural and Food Chemistry 2005 53 10074 10079 10.1021/jf0517703 16366697

[B58] Broadhurst CL Polansky MM Anderson RA Insulin-like activity of culinary and medicinal plant aqueous extracts in vitro Journal of Agricultural and Food Chemistry 2000 48 849 852 10.1021/jf9904517 10725162

[B59] Oswa T Uritany I, Garcia VV, Mendoza EM Novel natural antioxidants for utilization in food and biological systems Post-harvest Biochemistry of Plant Food Materials in the Tropics 1994 Tokyo: Japan Scientific Society Press 241 251

[B60] Ferguson RL Philpott M Karunasinghe N Dietary cancer and prevention using antimutagens Toxicology 2004 198 147 159 10.1016/j.tox.2004.01.035 15138038

[B61] Ikuma NEM Passoni HM Biso IF Longo CM Cardoso PRC Campaner SL Varanda AE Investigation of genotoxic and antigenotoxic activities of *Melampodium divaricatum *in *Salmonella typhimurium* Toxicology In Vitro 2006 20 361 366 10.1016/j.tiv.2005.08.012 16182509

[B62] Vundac VB Brantner AH Plazibat M Content of polyphenolic constituents and antioxidant activity of some Stachys taxa Food Chem 2007 104 1277 1281 10.1016/j.foodchem.2007.01.036

[B63] Skandrani I Bouhlel I Limem I Boubaker J Bhouri W Neffati A Ben Sghaier M Kilani S Ghedira K Ghedira-Chekir L *Moricandia arvensis *extracts protect against DNA damage, mutagenesis in bacteria system and scavenge the superoxide anion Toxicology in Vitro 2009 23 166 175 10.1016/j.tiv.2008.10.010 19015021

